# Auditory N1 event-related potential amplitude is predictive of serum concentration of BPN14770 in fragile X syndrome

**DOI:** 10.1186/s13229-024-00626-0

**Published:** 2024-11-02

**Authors:** Jordan E. Norris, Elizabeth M. Berry-Kravis, Mark D. Harnett, Scott A. Reines, Melody A. Reese, Abigail H. Outterson, Claire Michalak, Jeremiah Furman, Mark E. Gurney, Lauren E. Ethridge

**Affiliations:** 1 Department of Psychology, University of Oklahoma, 455 W. Lindsey Street, Dale Hall Tower, Room 705, Norman, OK 73019-2007 USA; 2 Department of Pediatrics, Neurological Sciences, and Biochemistry, Rush University Medical Center, Chicago, IL USA; 3 Tetra Therapeutics, Grand Rapids, MI USA; 4 Department of Anesthesiology, Duke University Medical Center, Durham, NC USA; 5 Department of Pediatrics, Section on Developmental and Behavioral Pediatrics, University of Oklahoma Health Sciences Center, Oklahoma City, OK USA

**Keywords:** Biomarker, Fragile X syndrome, Zatolmilast, EEG, Pharmacokinetics

## Abstract

Fragile X syndrome (FXS) is a rare neurodevelopmental disorder caused by a CGG repeat expansion ≥ 200 repeats in 5’ untranslated region of the FMR1 gene, leading to intellectual disability and cognitive difficulties, including in the domain of communication. A recent phase 2a clinical trial testing BPN14770, a phosphodiesterase 4D inhibitor, showed improved cognition in 30 adult males with FXS on drug relative to placebo. The initial study found significant improvements in clinical measures assessing cognition, language, and daily functioning in addition to marginal improvements in electroencephalography (EEG) results for the amplitude of the N1 event-related potential (ERP) component. These EEG results suggest BPN14770 improved neural hyperexcitability in FXS. The current study investigated the relationship between BPN14770 pharmacokinetics and the amplitude of the N1 ERP component from the initial data. Consistent with the original group-level finding post-period 1 of the study, participants who received BPN14770 in period 1 showed a significant correlation between N1 amplitude and serum concentration of BPN14770 measured at the end of period 1. These findings strengthen the validity of the original result, indicating that BPN14770 improves cognitive performance by modulating neural hyperexcitability. This study represents the first report of a significant correlation between a reliably abnormal EEG marker and serum concentration of a novel pharmaceutical in FXS.

## Background

Fragile X syndrome (FXS) is a rare neurodevelopmental disorder caused by a repeat expansion of > 200 CGG repeats in the 5’ untranslated region of the *FMR1* gene located on the X chromosome (Santoro et al. 2012; Straub et al. 2023). Clinical features of FXS include high rates of intellectual disability, anxiety, and difficulties with executive function. Cognitive features of FXS, including communication difficulties, are often cited as among the most distressing for individuals with FXS and their families with no treatment currently existing for ameliorating cognitive symptoms (Weber et al. 2019).

A recent phase 2 clinical trial assessing BPN14770 (now Zatolmilast), a first-in-class phosphodiesterase 4D (PDE4D) inhibitor, in adult males with FXS demonstrated cognitive improvements on the performance-based NIH Toolbox Cognitive Battery and in caregiver reports of language and daily functioning (Berry-Kravis et al. 2021). BPN14770 works to increase cyclic AMP (cAMP) levels by reducing phosphodiesterase activity (Gurney et al. 2019). The a priori secondary physiological measures included electroencephalography (EEG) which measured brain activity during an auditory habituation task. Specifically, the amplitude of the N1 event related potential (ERP) component was assessed and demonstrated marginal reductions suggesting improvements in neural hyperexcitability (Berry-Kravis et al. 2021). Generally, individuals with FXS exhibit increased neural responses to auditory stimuli compared to typically developed controls with the consensus that heighted neural responses to auditory stimulation reflects overall neural hyperexcitability as well as increased sensory sensitivity (Contractor et al. 2015; Ethridge et al. 2019). The habituation task primarily measures cortical hyper-excitability via absolute ERP amplitudes, and the ability to adapt cortical responses to repeated stimulation. While the reduced habituation phenotype itself has been recently found to be more variable at the individual level in FXS, elevated N1 amplitudes, indexing neural hyperexcitability, in this task are a robust phenotype in FXS that are associated with sensory sensitivity, auditory behavioral task performance, and autistic characteristics (see Ethridge et al. (2019) for more in-depth commentary). While BPN14770 does not target mechanisms related directly to neural hyperexcitability, a PDE4D inhibitor may support neural network organization by improving long-term potentiation through improved cAMP signaling (Mierau et al. 2017).

Given the validity of the EEG findings, the N1 amplitude outcomes provide a physiological bridge between known molecular mechanisms of BPN14770 and improvements in clinical outcomes. However, data loss in the habituation task (i.e., inability to collect data at a study visit or unusable data files due to artifact) resulted in reduced power to detect effects thus raising the question of whether the marginal efficacy findings for the N1 ERP reflected true reductions in neural hyperexcitability. To confirm validity of the finding, N1 amplitude reductions were assessed against plasma BPN14770 levels from pharmacokinetic assessment at the end of period 1.

## Methods

Participants were 30 males (age 18–41 years, *M* = 31.63, *SD* = 7.32; IQ 24.63–66.19, *M* = 42.78, *SD* = 11.6) with FXS participating in a single-center, phase 2 clinical trial assessing the efficacy and safety of BPN14770 (ClinicalTrials.gov identifier: NCT03569631, registration date: June 26, 2018). All participants or their legal guardians signed informed consent which included consent for EEG data collection. All study procedures were approved by the Institutional Review Board at Rush University Medical Center; EEG analysis and data management procedures were additionally approved by the IRB at University of Oklahoma.

## Procedure

### Habituation task

**Fig. 1:**
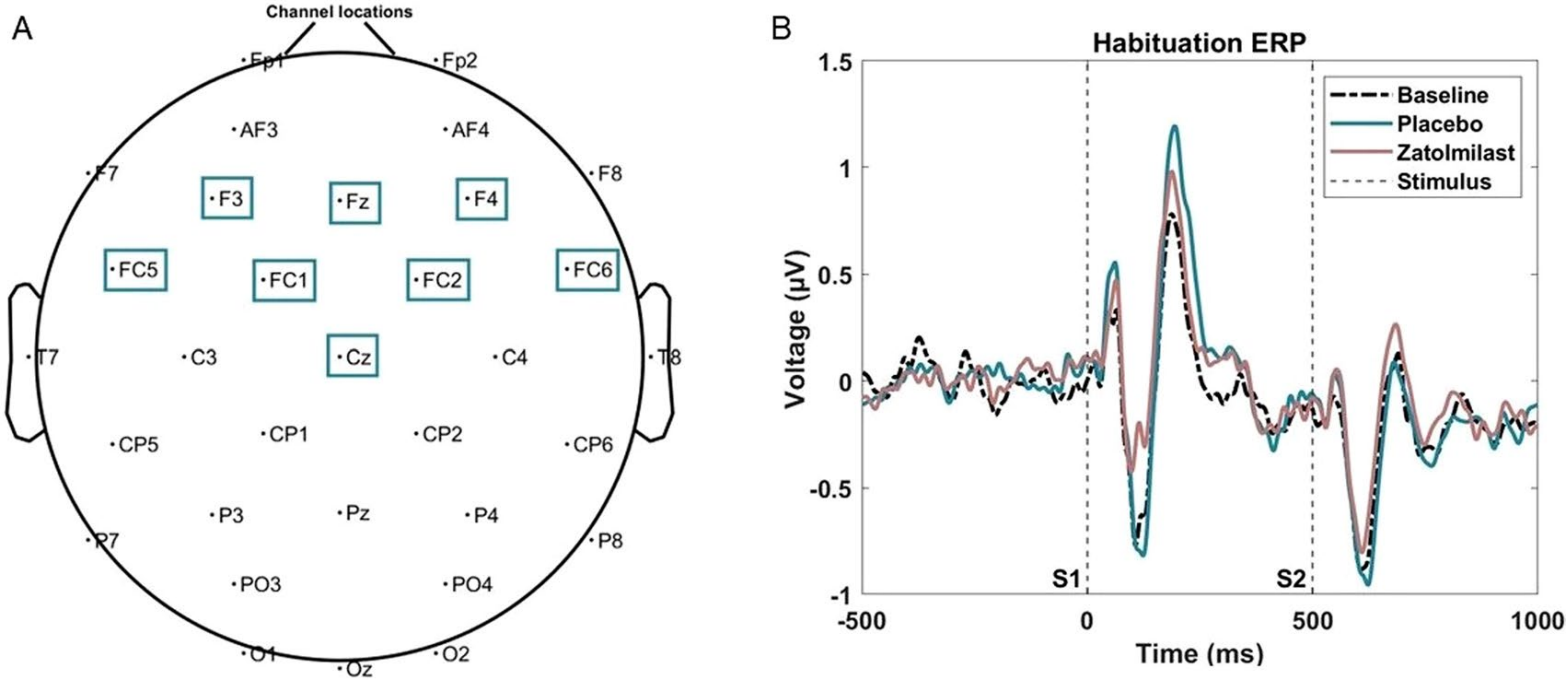
**A** Channel map for all 32 electrodes of the 32-channel BioSemi Cap with the frontocentral channels denoted by boxes. **B** The ERP waveform (*N* = 22 BPN14470 and *N* = 23 placebo participants) for both stimuli in the habituation pair from Berry-Kravis et al. (2021). Stimuli are identified by dashed vertical lines where S1 is the first stimulus and S2 is the second stimulus

The auditory habituation task consisted of 150 paired 50-ms white-noise bursts with a 500 ms interstimulus-onset interval. Each stimulus train was separated by a 4000 ms inter-trial interval for a total participation time of 11.5 min. Event related potential (ERP) values were measured at baseline, crossover (i.e., end of period 1), and again at the end of trial (i.e., end of period 2). The N1 amplitude was calculated for both stimuli in the habituation pair and defined as the most negative peak between 50 and 200 ms post-stimulus using a frontocentral cluster of EEG channels (Fig. 1; also depicting the original ERP figure with stimulus markers).

### EEG recording and preprocessing

EEG data were continuously recorded and digitized at 512 Hz using a 32-channel BioSemi ActiveTwo system (BioSemi) with a 5th order Bessel anti-aliasing filter at 200 Hz. Sensors were all active referenced to a driven right leg feedback loop between the central zenith (Cz) electrode located centrally at the top of the head and a central posterior ground electrode during recording. Data were inspected offline, resampled to 500 Hz, and preprocessed to remove artifacts prior to analysis using MATLAB 2018. In order, (1) data were digitally filtered offline from 0.5 to 100 Hz with a 57–63 Hz notch, (2) bad channels were visually inspected and interpolated with no more than ~ 5% of sensors interpolated (max of 2 channels), (3) segments with high artifact contamination (i.e., large movement-related artifacts) were manually rejected, (4) data were then submitted to independent component analysis (ICA) for further artifact correction, and (5) re-referenced to the average of all channels (Delorme and Makeig 2004).

### Pharmacokinetics

Pharmacokinetic (PK) samples were collected at baseline and at the end of each crossover arm to confirm the study drug was present when expected (Berry-Kravis et al. 2021). BPN14770 has a half-life of between 8 and 10 h (Zhang et al. 2018). Per protocol, the final dose was administered the morning of the post-period 1 study visit where blood draws and EEG assessments were performed, meaning BPN14770 was at steady state during both assessments. The EEG assessments occurred during the late morning or early afternoon for all participants. The original study did not have a sufficient wash-out period and therefore the main analyses were conducted on data from the end of period 1 between subjects (i.e., visit at study crossover). As a result, the post-period 1 PK value variable has 0 ng ml^−1^ BPN14770 if the participant received placebo during period 1.

### Statistical analysis

A Spearman rank correlation analysis was conducted to determine whether PK values correspond to a reduction in the N1 amplitude to the first stimulus in the habituation pair which demonstrated marginal significance in the original analysis of the EEG N1 ERP component (see Fig. 1 or Berry-Kravis et al. (2021). The correlation with the raw N1 amplitude was selected because it best matches the original analysis. Correlations were run separately assuming an alpha level of 0.05 with marginal effects defined as alpha level less than 0.10: (1) including both placebo and treatment groups, and (2) including just the treatment group, to account for inherent baseline variability in N1 amplitude across participants that may skew correlation values when all individuals in the placebo group have a PK value of 0 ng ml^−1^. Additional exploratory correlations were run to assess PK relationships with the first stimulus in the habituation pair where baseline N1 amplitude values were subtracted out, from here out referred to as baseline adjusted or change from baseline.

## Results

**Fig. 2:**
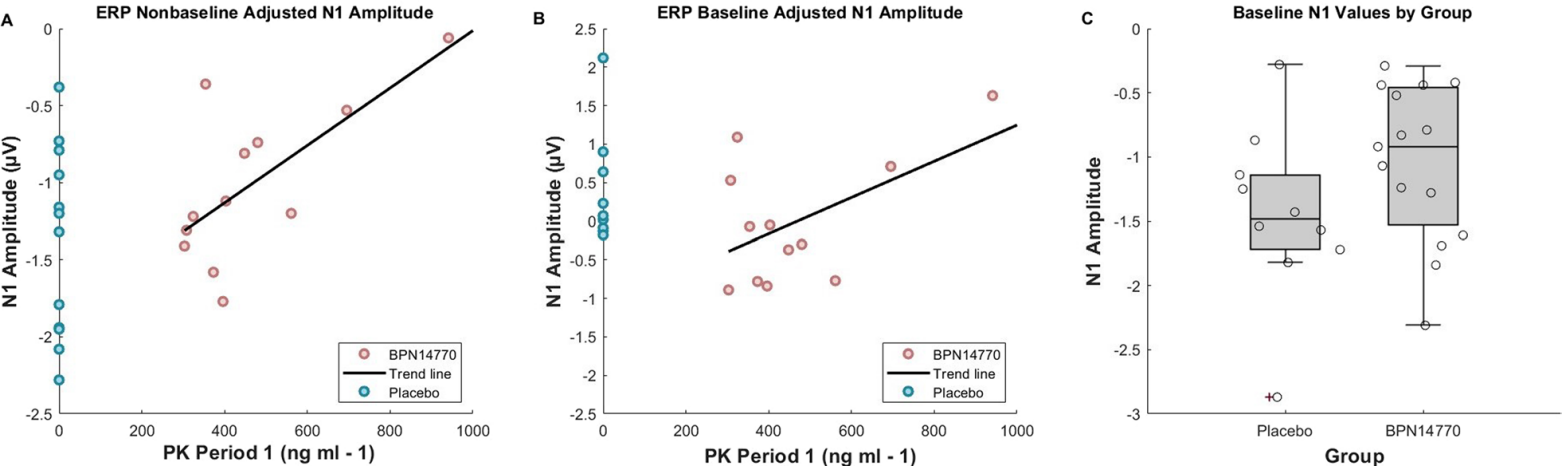
*Post-Period 1 PK – N1 Correlation Plot for the First ERP Peak. ***A** Correlation plot for post-period 1 PK values and N1 amplitude. All PK values at 0 are individuals who received placebo during period 1. The trend line reflects the relationship for the Zatolmilast group only. **B** Correlation plot for N1 amplitude values change from baseline and post-period 1 PK values. The trend line reflects the relationship for the Zatolmilast group only. Starting values were all negative, so negative values are not representative of participants having more negative N1 amplitudes on BPN14770 **C** Box plot with individual datapoints for the baseline N1 amplitude values between groups to show the spread of the N1 amplitude for individuals who went on placebo compared to those who went on BPN14770

Including both placebo and treatment groups in the analysis, there was a marginally significant positive correlation between the post-period 1 PK values and the N1 amplitude to the first stimulus (rho = 0.396, *p* = .055; *N* = 24 valid). The follow up correlation analysis run on only those in period 1 who received BPN14770 resulted in a statistically significant positive relationship between PK values and the N1 amplitude for stimulus 1 for those on BPN14770 during period 1 (rho = 0.608, *p* = 0.036; *N* = 12 valid; Fig. 2A) and raises confidence in the trend observed in the initial correlation analysis with those on placebo during period 1 included. When measured as a change value from baseline, there is not a relationship between N1 amplitude and PK values (rho = − 0.091, *p* = 0.696; *N* = 21 valid). The follow up analysis run on those who received BPN14770 in period 1 was also not significant but was increased compared to when the placebo values were included (rho = 0.308, *p* = 0.331; 12 valid; Fig. 2B) and visually follows a similar trend to the raw N1 amplitude correlation plot, in that, except for two individuals whose N1 amplitudes were responsive with lower serum concentrations, individuals with higher serum concentrations of BPN14770 show larger changes in N1 amplitude from baseline. It is important to note that the N1 ERP amplitude is a negative value, so positive correlations indicate a *reduction* in N1 amplitude with increased serum concentration of BPN14770.

## Discussion

Individuals with FXS tend to exhibit increased neural responses reflected by increased ERP amplitudes to auditory stimuli compared to typically developed individuals, likely due to neural hyperexcitability (Ethridge et al. 2016, 2019; Tempio et al. 2023). The original results from the phase 2a clinical trial suggested BPN14770 reduced the absolute N1 ERP component amplitude to the first stimulus in a habituation pair. Given the N1 is a negative-going ERP component, the positive correlation presented in the current study indicates a decrease in negative amplitude with BPN14770, which is an improvement that corresponds to a decreased neural response to the stimulus. Figure 2A demonstrates the expanded negative range of ERP values for the placebo group, highlighting the group-level shift in ERP amplitudes toward smaller values (i.e., range reduction), as well as the relationship between BPN14770 serum concentration and decreases in N1 amplitude. The individual with the largest plasma concentration of BPN14770 shows a nearly zero-amplitude value for N1, suggesting a potential upper limit to effective dosing for this individual for maintaining the N1 amplitude within typical levels. Additionally, it is important to note that, with the exception of two individuals, the majority of individuals in the placebo group showed change from baseline values close to zero (Fig. 2B). Despite the non-significant correlation between N1 amplitude change from baseline and PK values, the baseline adjusted BPN14770 values appear to maintain a similar trend to that noted in Fig. 2A. Baseline ERP values introduce another level of variability, notable in Fig. 2C, which reduces statistical power. Although absolute N1 amplitudes at a group level tends to follow known patterns in FXS (e.g. higher N1 values overall (Ethridge et al. 2019), and reduced N1 values at the group level from baseline and placebo for BPN14770 (Berry-Kravis et al. 2021) absolute amplitude at the individual level and timepoint can be influenced by a number of factors, such as impedances at the scalp, cap placement, and number of clean trials per run. This small increase in potential error variability compounds when adding additional timepoints to compute change values, which can reduce the power of the effect in small sample size studies such as these. Both findings highlight the necessity of a replication with Phase 3 data.

Despite clear limitations, the current results raise confidence in the validity of the original, marginal ERP amplitude group-level result in favor of BPN14770 improvements. Confirmation of prior results lends support to the conclusion that BPN14770 reduces neural hyperexcitability during stimulus processing. Variations in the auditory N1 ERP have been associated with language and communication as well as sensory outcomes in FXS (Ethridge et al. 2016, 2019; Schmitt et al. 2020) the results have implications for language processing and may underlie the noted improvements in clinical outcomes from the original study (Berry-Kravis et al. 2021).

### Limitations

Limitations included the small sample size consistent with Phase 2 studies, expected data loss in EEG data due to movement which further reduced statistical power, and carry-over effects in placebo measures from period 2. While the correlation supports the original findings, and suggests that the effect was present but underpowered, both post-period 1 analyses within the BPN14770 group reflect only 12 individuals. Nevertheless, this study represents the first report of significant correlation between a reliably abnormal EEG marker and serum concentration of a novel pharmaceutical in FXS. The limited sample size may inflate the effect of sources of variability. Specifically, differences in cAMP levels may account for placebo variability but cAMP levels were not assayed for this study as BPN14770 inhibits PDE4D, a predominantly brain specific enzyme (with a goal of avoiding the peripheral side effects of this class of drugs) and, therefore, it was assumed that level in blood after drug initiation would not be a useful marker of activity. Thus-we cannot control for cAMP levels to determine target engagement. The most evident limitation is the statistically non-significant relationship between baseline adjusted N1 values and PK but, again, those differences could reflect natural variability in the baseline ERP values due to non-neural sources. The N1 amplitude will be assessed as part of the ongoing phase 3 trial testing BPN14770 in a larger sample which will provide a sufficiently powered statistical model for assessing N1 amplitude improvement with BPN14770 treatment and the relationship it may share with serum BPN14770 concentrations.

## Data Availability

All data generated or analyzed during this study are included in this published article. All data points reported in the analyses are presented in Fig. 2.

## Abbreviations

FXS: Fragile X syndrome
FMR1: Fragile X messenger ribonucleoprotein 1
EEG: Electroencephalography
ERP: Event-related potential
PK: Pharmacokinetics
NIH: National Institutes of Health
PDE4D: Phosphodiesterase 4D
